# Case Report: Energy- and Nutrient-Dense Formula for Growth Faltering: A Report of Two Cases From India

**DOI:** 10.3389/fnut.2021.588177

**Published:** 2021-02-26

**Authors:** Zahid Ul Kareem, Suresh Kumar Panuganti, Shikha Bhatia

**Affiliations:** ^1^Department of Pediatric Critical Care, Ankura Children's Hospital, Hyderabad, India; ^2^Department of Pediatric Critical Care, NICE Hospital for Women, Newborns and Children, Hyderabad, India; ^3^Healthcare Nutrition Science, Nutricia International Pvt. Ltd., Mumbai, India

**Keywords:** ENDF, India, infant, growth faltering, PICU, nutritional management, catchup growth

## Abstract

Infants hospitalized for critical illnesses are at a high risk of undernutrition because of increased nutrient requirements (due to increased metabolism) and decreased nutrient intake (due to disease-related problems such as anorexia or feeding difficulties). This can result in a slowing down of their normal growth, referred to as “growth faltering.” Appropriate nutritional management of these infants is extremely important to avoid long-term adverse outcomes. Administration of energy- and nutrient-dense formula (ENDF; 100 kcal energy and 2.6 g protein per 100 mL, with added micronutrients) can be an effective means of increasing the nutrient and energy intake of these children. Despite the high prevalence of undernutrition and growth faltering among pediatric patients in India, there is a paucity of literature on the use of ENDF in Indian infants. Herein, we report the successful use of ENDF for the nutritional management of two infants hospitalized for growth faltering because of severe upper airway obstruction. The aim of nutritional management was to achieve satisfactory weight gain, which can lead to spontaneous resolution of upper airway obstruction. ENDF was initially administered to provide 50–100 kcal/kg/day, and the dose was gradually increased to 160–185 kcal/kg/day. Both infants tolerated the formula well and showed satisfactory weight gain. These cases clearly demonstrate that early administration of ENDF is an effective means of increasing nutrient and energy intake of critically ill infants, thereby facilitating catchup growth, without any notable adverse effects.

## Introduction

Undernutrition is a major concern among hospitalized pediatric patients across the globe. The reported prevalence of undernutrition among this population ranges from 5 to 50% and is, in general, higher in developing countries such as India than in developed countries (1–9). An Indian cross sectional study, reported that a large proportion (39.8%) of hospitalized children (<5 years) were diagnosed as malnourished (10). The coexistence of disease and undernutrition exerts several adverse effects on the growth and development of pediatric patients, with each intensifying the effects of the other. Critically ill infants have increased metabolic requirements, and hence, are at a higher risk of undernutrition (11). These infants usually also have decreased nutrient intake because of anorexia, feeding difficulties, or the effect of medications (12). In addition, both acute illnesses (e.g., trauma, burns, and infections) and chronic diseases (e.g., cystic fibrosis, congenital heart disease) are usually associated with increased nutrient loss and altered nutrient utilization (13). Conversely, undernutrition in hospitalized pediatric patients has been shown to be associated with delayed recovery (12), increased length of hospital stay, higher mortality and complication rates, and decreased quality of life (14–16). Hence, adequate nutritional support for these patients is of utmost importance.

Absence of appropriate nutritional interventions for these patients increases the risk of growth faltering, previously also referred to as “failure to thrive.” Growth faltering is a condition in which the infant's rate of weight gain is lower than that expected for his/her age and sex (17). The following criteria are used most frequently to diagnose growth faltering in an infant/child: (a) weight falling through two major centile lines on standard weight charts, (b) weight falling below the 3rd centile for age and sex, or (c) weight for height <2 standard deviations below the mean for age and sex. If left unchecked, growth faltering can have serious long-term consequences. Not only is immune dysfunction common in these infants and children, they are also at a high risk of inadequate cognitive development and impaired intellectual, social, and psychological functioning in the long term (18).

The first step in the treatment of growth faltering due to an underlying medical condition is the treatment of the condition itself (19). In addition, timely initiation of nutritional management is also extremely important (20). To facilitate catchup growth, infants with growth faltering need to be provided 150% of the recommended daily caloric intake for their expected (and not actual) weight for age (19). However, energy supplementation without simultaneous provision of proteins and micronutrients, as was the norm previously, is a flawed practice and inhibits catchup growth (21). Accordingly, in 2007, a Joint WHO/FAO/UNU Expert Consultation issued a guideline that in order to optimize catchup growth of lean and fat mass, 8.9–11.5% of energy should be provided as protein (22). The joint guidelines issued by the Society of Critical Care Medicine (SCCM) and American Society for Parenteral and Enteral Nutrition (ASPEN) state that a minimum protein intake of 1.5 g/kg/day is required to prevent cumulative protein deficits in critically ill children. This requirement can even go up to 2.5–3 g/kg/day in specific patient populations, such as infants and children hospitalized for bronchiolitis or other causes of respiratory failure requiring mechanical ventilation (23).

Energy- and nutrient-dense formulae (ENDF) are an effective solution to meet the energy and nutrient requirements of infants with growth faltering. Compared to standard infant formulae, they contain up to 52% more energy (420 kJ or 100 kcal per 100 mL), up to 73% more protein (2.6 g protein per 100 mL; 10.4% of the energy provided by the protein component), and 50% higher concentrations (range, 19–111%) of most micronutrients and vitamins (21) (Table 1). Their efficacy in facilitating catchup growth in infants with growth faltering, as well as their tolerability, has been demonstrated in several studies (21, 24–30). However, to our knowledge, there has been no report of their use in the Indian population. Herein, we report two cases from India involving the successful use of an ENDF for nutritional management of infants hospitalized with severe growth faltering due to underlying disease.

**Table 1:** Energy and nutrient dense formula, ingredient list.

**Nutrients**	**Unit**	**Approximate Composition**	
		**per 100g**	**per 100 ml**
**Energy**	kcal	505	100
	kj	2114	418.6
**Fat**	g	26.0	5.15
Milk fat	g	12.5	2.48
Saturated fatty acids	g	10.0	1.98
Mono-unsaturated Fatty acids	g	11.0	2.17
Poly-unsaturated fatty acids	g	4.1	0.82
Trans Fatty acids	g	0.5	0.09
Cholesterol	mg	36	7.13
α -Linolenic acid (omega 3)	mg	340	67.32
Linoleic acid (omega 6)	mg	3400	673.2
Oleic Acid	mg	10.3	2.04
**Protein**	g	13	2.57
60% Whey protein	g	7.8	1.54
40% Casein	g	5.2	1.03
**Carbohydrate**	g	54.8	10.85
Sugar	g	0	0.0
Taurine	mg	41	8.12
Carnitine	mg	8.5	1.68
**Vitamins**			
Vitamin A	mcg RE	410	81.18
Vitamin D	mcg	9	1.78
Vitamin E	mgT E	6.7	1.33
Vitamin K	mcg	38.0	7.52
Thiamine	mcg	526	104.15
Riboflavin	mcg	760	150.48
Niacin	mg	5.5	1.09
Vitamin B6	mcg	350	69.3
Folic acid	mcg	79	15.64
Pantothenic acid	mg	2.4	0.48
Biotin	mcg	20	3.96
Vitamin B12	mcg	1.5	0.03
Vitamin C	mg	60.0	11.88
Choline	mg	84	16.63
**Minerals**			
Calcium	mg	500	99
Phosphorus	mg	290	57.42
Magnesium	mg	38	7.52
Sodium	mg	180	35.64
Potassium	mg	460	91.08
Chloride	mg	300	59.4
Iron	mg	7.0	1.39
Zinc	mg	3.8	0.75
Copper	mcg	350	69.3
Iodine	mcg	76	15.05
Manganese	mcg	80	15.84
Selenium	mcg	20	3.96
**Nucleotides**	mg	18	3.56

## Case Description

### Case 1

A 7-month-old male child, born normally at full term with no dysmorphic features, was admitted to the hospital because of a severe attack of bronchopneumonia. He had been suffering from severe upper airway obstruction since birth, resulting in poor oral feeding. He had a history of multiple hospitalizations. He had undergone surgery for bilateral inguinal hernia shortly after birth (postnatal day 9), with no postoperative complications. However, a few days after discharge, his parents had noticed recurrent noisy breathing, which increased with time. He was also suffering from apneic attacks with diaphoresis during feeding; hence, oral feeding was not possible. The child was readmitted to the hospital but due to the impossibility of oral feeding, he was discharged on nasogastric feeding (regular infant milk formula with medium-chain triglyceride oil as no ENDF was available in India at that time). After discharge, he had to be hospitalized repeatedly because of aspiration pneumonia. Laryngoplasty was performed but oral feeding was still not possible because of residual narrowing, and the child was once again discharged on nasogastric feeding with standard infant formula plus medium-chain triglyceride oil. However, no weight gain was observed up to 3 months of age.

At the time of the current hospitalization, the child was on nasogastric feeding. His weight was 5.130 kg, height 61 cm, head circumference 43 cm, and mid-upperarm circumference (MUAC) was 12.5 cm. No abnormalities were observed on chest radiography, chest computed tomography (CT), echocardiography, brain magnetic resonance imaging, barium meal, and milk scan. Blood pressure and serum electrolyte levels were also normal. However, bronchoscopy showed signs of laryngomalacia and CT of the paranasal sinuses revealed narrowing and near occlusion (II degree) of the oropharynx. Posteriorly placed adenoids were seen. Based on these observations and the fact that the child's weight was less than the 3rd percentile on standardized WHO growth charts, he was diagnosed with growth faltering due to severe laryngomalacia.

The patient was admitted to the pediatric intensive care unit (PICU) and kept there for 3 weeks. An ENT specialist was consulted, who advised that tracheostomy be performed provided the child showed good oropharyngeal coordination and satisfactory weight gain. However, the child was not able to gain weight. Moreover, feed volume could not be increased to facilitate weight gain as gastroesophageal reflux disease was suspected. Thus, it was decided to initiate nutritional management with ENDF. The aim was to achieve enough weight gain to perform tracheostomy without any adverse events as satisfactory weight gain can lead to spontaneous resolution of upper airway obstruction, as well as better immunity and normal neurodevelopmental outcomes. Initially, the child was administered a 60–70 mL feed of ENDF every 3 h, adding up to a total energy intake of 100 kcal/kg/day. Gradually, under the pediatrician's guidance and over a period of 1 week, the intake was increased to 160–170 kcal/kg/day. The feed was well-tolerated, with no signs of diarrhea, flatulence, or vomiting. The child gained 400 g over 2 weeks and weighed 5.530 kg at the end of the 3-week PICU stay. This was the maximum growth velocity (30 g/day) ever achieved in the child's life. As a result, tracheostomy was performed successfully.

**Figure 1:**
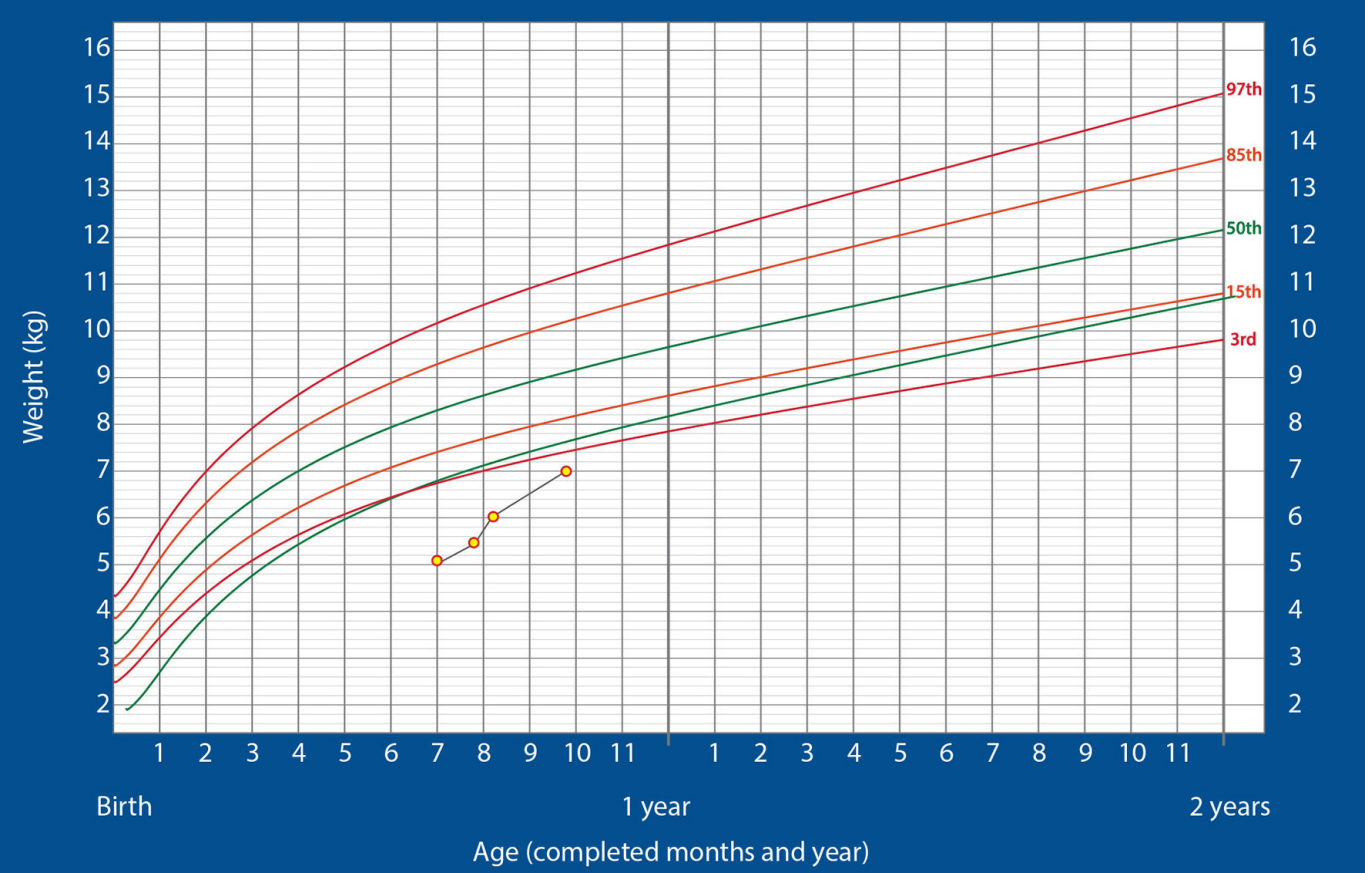
Changes in the weight of the infant over time, plotted on the standardized WHO weight-for-age growth chart for boys (Case 1).

At this point, the infant was shifted to the ward and kept there for 2 additional days before being discharged on nasogastric feeding with 160–170 kcal/kg/day of ENDF. At discharge, he weighed 5.445 kg. The drop in weight was because of the posterior rhinoplasty he had to undergo during the third week of hospitalization. After discharge, the child gained weight rapidly, reaching 6 kg at the 2-week follow up. He did not show any signs of intolerance to the formula and was thriving well. Finally, at 8 weeks after discharge, when the child was weighing 7 kg, the nasogastric tube was successfully removed. After the removal of the tube, the oral feeding was continued with ENDF and home-made foods. Alternate feeds were given between ENDF formula and home-made diet as recommended for age (7 months) wherein ENDF accounted for 360–480 kcals/day of the total caloric intake which composed of 3-4 feeds, the child gradually developed tolerance for semi solid home cooked foods. Even after 8 weeks post discharge, patient regularly visited for follow up and was on infant formula and home cooked meals till the age of 12 months, meeting his recommended daily allowance as designed for Indians. The child's growth chart is shown in Figure 1. The child's parents were pretty satisfied and credited the ENDF formula based nutritional intervention, as the importance of weight gain was emphasized to them by many specialist during their past hospitalizations.

### Case 2

A male child aged 1 year and 20 days who was immunized upto date was admitted to the hospital with complaints of fever and breathlessness that had persisted for a week. At the time of admission, he was febrile, with severe respiratory distress (clinical respiratory score of eight) and bilateral crackles, and was on gastrostomy feeds. His weight was 8 kg, height 76 cm, head circumference 45 cm, and MUAC was 13 cm. Chest radiography revealed haziness in the bilateral lung fields, indicating pneumonia. Hemoglobin (11.3 g/dL), platelets, serum C-reactive protein (2.4 mg/L), and packed cell volume (31.3%) were all within normal limits. However, the child's leucocyte count was elevated (19,400 cells/μL)—a sign of severe infection—and blood culture revealed the presence of *Klebsiella pneumoniae*. Based on the clinical and laboratory findings, a primary diagnosis of severe bronchopneumonia with sepsis was made. Since the child's weight was less than the 3rd percentile on WHO standardized growth charts, he was also diagnosed with growth faltering.

As the child was in respiratory failure at the time of admission, he was immediately shifted to the PICU for mechanical ventilation with high-frequency oscillation. While on the ventilator, he had a cardiac arrest, and also developed seizures and acute kidney injury. At this stage, total parenteral nutrition was started. The child remained in the PICU for 12 days, after which he was taken off the ventilator and shifted to the ward. However, after extubation, he showed neurological deficit (minimal) and poor oral feeding. He had lost 1.5 kg over the past 12 days. Hence, administration of ENDF via gastrostomy tube was initiated as the sole source of nutrition at a dose of 50 kcal/kg/day. As the child tolerated the formula well, the dose was gradually increased to 185 kcal/kg/day.

**Figure 2:**
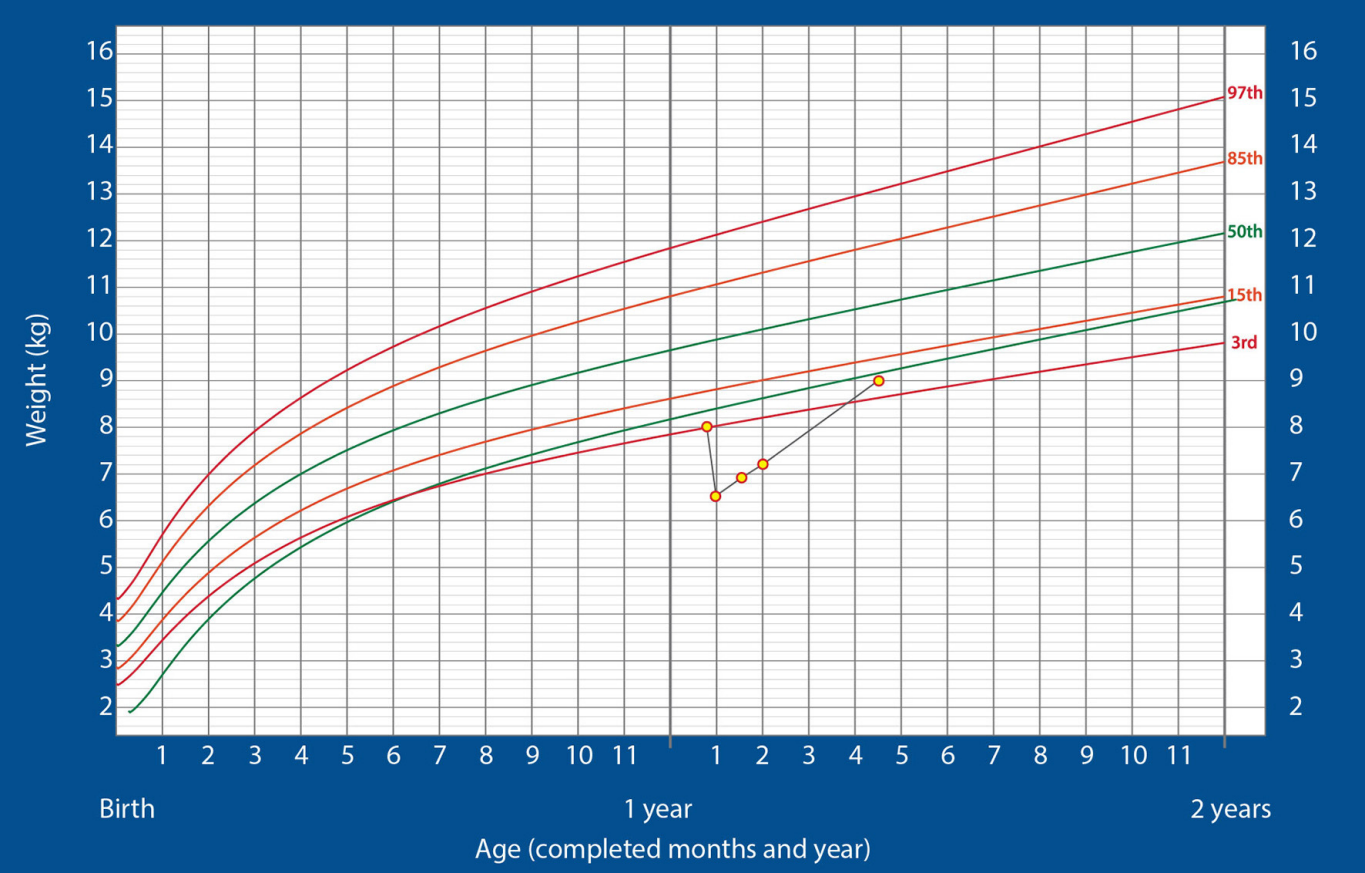
Changes in the weight of the infant over time, plotted on the standardized WHO weight-for-age growth chart for boys (Case 2).

The child gained 400 g over the next 10 days and weighed 6.9 kg at the end of 23 days of hospitalization. At this stage, he showed no neurological deficit and was active. Hence, he was discharged and continued on ENDF feeds through gastrostomy tube (185 kcal/kg/day) at home. At the 2-week follow-up, the child's weight was 7.2 kg and he was doing well. Hence, minimal oral feeding was started with the intention of removing the gastrostomy tube at the first follow-up. The child continued alternate feeds between ENDF and home-cooked meals and ENDF was stopped once the child reached 9 kg of weight at 3 months post discharge and was thriving well on the WHO growth charts, post which child was completely on home cooked meals and did not need close follow up. The child demonstrated a substantially high post-discharge rate of weight gain (700 g/month). The growth chart is shown in Figure 2. The child's parents were happy and have noticed the weight gain significantly higher as compared to regular feeds. They were profoundly thankful for the ENDF formula.

## Discussion

Despite the high prevalence of undernutrition in India, evidence on the use of ENDF in Indian infants is scarce. To our knowledge, ours is the first report from India describing the use of ENDF to promote catchup growth in infants with growth faltering, caused by laryngomalacia in one case and bronchopneumonia complicated with sepsis in the other. The airway obstruction in laryngomalacia makes it difficult for the infant to co-ordinate the suck-swallow-breathe sequence required for feeding, and the increased metabolic requirements for feeding eventually result in weight loss (31). Likewise, severe pneumonia can impact the digestive, circulatory or respiratory systems which may result in growth faltering (32). Both infants showed satisfactory weight gain with enterally administered ENDF, without major adverse effects.

Nutritional management of critically ill infants with growth faltering has always been a challenge. Before the advent of ENDF, several strategies were employed to increase the nutrient intake of these infants. Since it was not always possible to increase feed volume, the energy content of standard infant formula was often increased by adding a glucose polymer and/or long-chain triglyceride emulsion. However, this technique increased only the energy intake and not intake of other nutrients essential for adequate catchup growth. Moreover, it reduced the energy derived from protein from 8 to 5.5%, thus disturbing the protein-energy ratio of the feed, which is of utmost importance to maintain adequate nitrogen balance (21). Deficiencies of minerals (e.g., zinc, iron) and vitamins (e.g., vitamin A) are known to cause growth faltering in children, and micronutrient supplementation has been shown to elicit positive growth responses (33). Inadequate intake of protein relative to the overall energy intake during catchup growth increases the deposition of adipose tissue relative to lean tissue, *i.e*., the weight gain is primarily in the form of fat. To sustain high rates of lean tissue deposition, the diet should have a high protein-energy ratio (34). Hence, nutritional management for growth faltering in infants should ensure adequate intake of protein and micronutrients, in addition to energy.

Another strategy employed to facilitate catchup growth was to concentrate standard infant formula to increase its nutrient density, for example, by adding 15 g of formula (instead of 13 g) to 100 mL of water. However, the increase in nutrient density achieved by this method was not sufficient for some infants. Concentrating the formula beyond this point caused a disproportionate increase in the concentration of some nutrients and also increased the renal solute load. Overconcentration or supplementation of infant formula could also increase its osmolality beyond the recommended limit of 400 mOsm/kg, which might cause osmotic diarrhea or necrotizing enterocolitis. In addition, both these techniques of feed manipulation carried a high risk of microbial contamination and mistakes during preparation, particularly if performed at home by parents or caregivers (21, 35).

The use of ENDF provides a solution to all the abovementioned challenges. These formulae provide a better protein-energy ratio, higher concentrations of micronutrients, and lower osmolality than energy-supplemented or concentrated infant formulae. Also, being a ready to use complete formula which doesn't need mixing of any additional supplements, the ENDF minimizes the risk of microbial contamination (33). The superiority of ENDF over energy-supplemented formula for nutritional management of infants with growth faltering due to underlying medical conditions has been demonstrated in a classic study by Clarke et al. They showed that although both formulae were well-tolerated and caused similar weight gain as well as energy intake, the use of ENDF was associated with significantly greater protein and micronutrient intakes. Moreover, infants receiving energy-supplemented formula showed a 50% drop in blood urea concentration, indicating a suboptimal protein-energy ratio in the formula, and a significant decrease in length z-score. In contrast, infants receiving ENDF maintained a normal blood urea concentration and did not show a significant decrease in length z-score (21).

Clarke et al.'s findings were corroborated by van Waardenburg et al. who compared the use of ENDF with that of standard infant formula in infants admitted to the PICU for respiratory failure due to respiratory syncytial virus bronchiolitis. Both groups of infants were compared over the first 5 days after admission to the PICU, and it was found that although both formulae were well-tolerated, infants receiving ENDF had higher cumulative nitrogen balance and cumulative energy balance. ENDF-fed infants achieved the population reference intakes for energy, protein, carbohydrates, and fat by day 3 itself, while in infants receiving standard infant formula, this was achieved only on day 5. In addition, levels of essential amino acids were below reference limits in infants receiving standard formula, but within reference limits in those receiving ENDF (25). The higher protein content of ENDF is of particular importance in the nutritional management of critically ill infants because critical illness is associated with increased protein breakdown, negative protein balance, and unfavorable clinical outcomes (26). The observation of higher essential amino acid levels in infants receiving ENDF in this study was significant because it is well-established that increase in the concentrations of essential amino acids, particularly branched-chain amino acids, stimulates muscle protein synthesis, suppresses protein breakdown, and improves the net protein balance (36, 37). Thus, administration of ENDF counteracts the adverse effects of critical illness in infants.

In line with these observations, de Betue et al. showed that ENDF feeding promoted protein anabolism in infants admitted to the PICU with viral bronchiolitis. Using an intravenous-enteral phenylalanine/tyrosine stable isotope method protocol, they showed that the higher protein balance in infants fed ENDF was achieved through a greater increase in protein synthesis than in protein breakdown relative to infants fed standard infant formula (26). The same study group subsequently showed that administration of ENDF also increased arginine appearance (compared to standard infant formula) in these infants, thereby increasing arginine availability for nitric oxide (NO) synthesis, independent of plasma arginine concentrations. Arginine plays important roles in wound healing, cell regeneration, immune function, and tissue perfusion, and NO is an important signaling molecule with multiple functions, including maintenance of microcirculation and tissue oxygenation. Increasing arginine availability and NO production is, thus, a target during critical illness, and it appears that the use of ENDF can facilitate this (27). The efficacy and tolerability of ENDF have been demonstrated in several other studies including diverse patient subgroups, for example, infants who have undergone congenital heart surgery (28, 38), those with a prolonged (≥7 days) PICU stay (29, 30), and those with complex disease (39). In these studies, ENDF use was found to be associated with feeding intolerance, manifesting as abdominal distention, diarrhea, and gastric retention. However, these adverse events were either very rare (29, 30) or easily treated with medication (28, 38). One limitation of ENDF observed in our practice is the risk of iron deficiency anemia because of the predominantly milk-based feeding (to achieve adequate calories); hence, we supplement ENDF with iron therapy.

It is crucial to note that the present case reports specifically highlights an effective nutritional management approach with ENDF for infants with growth faltering because of upper airway obstruction. At present, we need more evidence to say that these formula can be given to all children and infants.

In summary, ENDF is useful in the nutritional management of infants with growth faltering and offers several benefits over standard infant formulae as well as energy-supplemented/concentrated formulae. Although we could not follow-up the infants over a long duration to assess their growth parameters and compliance with ENDF feeding, the cases described herein suggest a positive effect of ENDF on recovery from disease-associated malnutrition. Breast milk may serve as the best source of nutrition for critically ill infants; however, in its absence, early administration of ENDF can ensure adequate energy and nutrient intake, thereby facilitating catchup growth, without any notable adverse effects. Since the ENDF formula is affordable to wide population (INR 500/- per 400 g), government aid in this case may not be required. The right dosage of the formula should be decided by the attending pediatrician or dietitian. This may vary from infant to infant and will depend on age, weight, nutritional status, tolerance, and the underlying condition. In general, the infants with increased energy needs will mostly require 110–160 kcal/kg/day (110–160 ml/kg/day of ENDF). ENDF also helps promote catchup growth after discharge, once there are no fluid restrictions. For compliance in the long run, regular patient visits and check-ups as per the direction of the physician may be required. Nevertheless, further research and long-term randomized trials are warranted to conclusively establish its utility in other critical illnesses as well as in the Indian scenario.

## Data Availability Statement

The original contributions presented in the study are included in the article/supplementary materials, further inquiries can be directed to the corresponding author/s.

## Ethics Statement

Written informed consent was obtained from the legal guardians of the patients for the publication of this case report.

## Author Contributions

SB conducted the literature search and drafted the manuscript. ZK and SP were involved in patient management and contributed to the acquisition of data and data interpretation. ZK, SP, and SB critically reviewed and revised the manuscript. All authors have read and approved the final version of the manuscript and given their approval for publication.

## Conflict of Interest

SB is employed with Danone Nutricia International Pvt. Ltd., Mumbai. ZK and SP confirm that they do not have any commercial or financial relationships that could be construed as a potential conflict of interest in relation to the publication of this report.
